# Overcoming Research Mistrust in African American Communities by Engaging Community Members as Research Team Members: Challenges and Opportunities

**DOI:** 10.1089/heq.2024.0050

**Published:** 2024-09-12

**Authors:** Melissa Ryan, Travaé Hardaway Griffith, Grace Okoro, Tiffany Osborne, Lori Brand Bateman, Janet M. Turan, Raegan W. Durant, Lece Webb, Mona N. Fouad, Gabriela R. Oates

**Affiliations:** ^1^ Heersink School of Medicine, University of Alabama at Birmingham, Birmingham, Alabama, USA.; ^2^ School of Public Health, University of Alabama at Birmingham, Birmingham, Alabama, USA.

**Keywords:** COVID-19, research mistrust, community engagement, community health workers, study recruitment

## Abstract

**Objectives:**

We aimed to understand factors surrounding COVID-19 testing in vulnerable urban and rural African American communities in Alabama, which are characterized by mistrust in medical research.

**Methods:**

To address widespread mistrust, we trained lay community members as research coordinators (Community Engagement Coordinators—CECs) and employed them for study recruitment and data collection. We then explored their experiences through group discussions and individual interviews.

**Results:**

Ten CECs (8 in Jefferson, 2 in Dallas County; 8 female, 2 male) completed 5 h of instructor-led training followed by virtual Collaborative Institutional Training Initiative (CITI) Human Subjects Training. In 11 weeks, CECs recruited 318 study participants and administered 303 surveys. After survey data collection was completed, CECs recruited survey respondents for participation in focus groups, enrolling 53 individuals. CECs continued their study engagement by reviewing developed study products.

**Conclusions:**

Engaging CECs as research personnel facilitated successful completion of planned enrollment with minimal missing data. Investing in communities by training and employing community members as study personnel can help overcome research mistrust and promote support for research and public health interventions.

## Introduction

Testing for the SARS CoV-2 virus, including new viral strains, remains a critical aspect of pandemic control even with the availability of COVID-19 vaccines. COVID-19 testing is particularly important in regions and populations with low vaccination rates, as it is a powerful tool to reduce the spread of COVID-19. Knowing their COVID-19 status can help people avoid spreading the infection to others. As of May 2023, only 7.7% of the total population and 9.6% of those age 18 years and older in the State of Alabama had received a bivalent booster dose, the second lowest in the continental United States.^1^ Although racial and ethnic disparities in the uptake of at least one COVID-19 vaccination dose have narrowed over time, African American people continue to have lower vaccination rates than other racial groups. For example, as of February 2023, 51% of African American individuals had received at least one vaccine dose, compared with 57% of White, 67% of Hispanic, 71% of Native Hawaiian and other Pacific Islander, 73% of Asian, and 78% of American Indian and Alaska Native counterparts.^1^ As of March 2023, African American individuals were only half as likely as White individuals to have received a vaccine booster dose.^1^

Considering the crucial role of COVID-19 testing as a pandemic control tool, our project, Reducing Ethical and Social Prejudicial Effects of COVID-19 Testing in Underserved Populations (RESPECT-UP), aimed to understand social, ethical, and behavioral factors surrounding COVID-19 testing in socioeconomically vulnerable urban and rural African American communities in Alabama. However, the study’s target population is characterized by high levels of mistrust in medical research,^2,3^ making study recruitment challenging. Research mistrust is defined as the belief that the interests of researchers and medical institutions supersede the interests of study participants, exemplified by withholding important information, imposing risks that outweighs the potential benefits, or using study data in a way that damages individuals or communities.^4^ The adverse consequences of such mistrust have been widely described in the literature. For example, research mistrust has been associated with reduced access to preventive services such as cancer screenings and influenza vaccinations, increased risk of chronic disease, and higher rates of disability and mortality.^3,5–10^

In the African American community, research mistrust can be traced back to an extensive history of medical maltreatment, including medical experimentation during slavery and, more recently, the U.S. Public Health Service Syphilis Study at Tuskegee.^11^ The legacy of mistreatment continues to impact the health care decisions of African Americans today, including their decisions regarding testing and vaccination for COVID-19.^12^ A study of African Americans who reside in low-income communities in Birmingham, Alabama, identified mistrust in COVID-19 messaging and health care providers as a major barrier to COVID-19 testing and vaccination in this population.^13^ Decline in engagement with the health care system, coupled with a decreased likelihood for referrals for COVID-19 testing, has contributed to stark disparities in COVID-19 testing, vaccination, infection, and health outcomes between African American and White people.^2,5,7^

The intersection of mistrust in medical research with health disparities experienced by vulnerable African American populations has created a unique dilemma for researchers in the area of minority health and health equity. To reduce disparities, addressing the mistrust has become imperative.^14–16^ Successful strategies for restoring the trust of African American people in medical science have involved various forms of community engagement, from community advisory boards and community health workers to community action plans and asset-based community development.^17–23^ Such participatory approaches have been powerful tools for addressing trust barriers, improving health outcomes among disadvantaged populations, and reducing racial and ethnic health disparities.

Building on the successful community health worker model,^24,25^ in this study, we developed the role of Community Engagement Coordinator (CEC). A CEC is a lay member of the target community who is trained in research with human subjects and used to recruit study participants and collect data. Our reasoning was that engaging CECs as research personnel to interact with potential study participants in the African American community would mitigate mistrust through better representation, facilitate effective and efficient study enrollment, and increase community support for COVID-19 research and interventions. The ultimate goal is improved health outcomes for African American people.

## Materials and Methods

### Study Population and Design

The RESPECT-UP study was implemented in two Alabama counties: Jefferson (urban) and Dallas (rural). Jefferson County is home to Birmingham, known for its critical role in the Civil Rights Movement. Dallas County, in the Black Belt Region, is home to Selma, known for the landmark Selma to Montgomery March, which led to the passage of the Voting Rights Act of 1965. In both counties, the study recruited African American people residing in Census tracts ranked in the top 10% (most vulnerable) of the CDC Social Vulnerability Index.^26^

A Community Advisory Board (CAB) of 12 representatives of community-based, faith-based, health care, media, and social service organizations in both counties provided guidance on intervention design and implementation and facilitated the recruitment of CECs.

Using a sequential explanatory mixed methods design, RESPECT-UP collected and analyzed quantitative data (a 149-question survey including required Tier 1 RADx-UP Common Data Elements and additional, project-specific questions) and qualitative data (focus groups and key informant interviews) to explore social, ethical, and behavioral factors surrounding COVID-19 testing in the context of vaccine availability. The study then developed and tested actionable strategies, in the form of toolkits tailored to various organizational and community contexts, to reduce inequities in COVID-19 testing. The recruitment and consent of study participants and the collection of survey data were carried out by as CECs.

### Recruitment of CECs

The study’s community engagement team reached out to our CAB and community partners in Jefferson and Dallas Counties (see acknowledgment section) and asked them to recommend motivated local residents who were devoted to and trusted by people in their communities and who were willing to undergo training and serve as CECs for the RESPECT-UP study. There were no other educational or experiential credentials required for CEC enrollment.

### Training of CECs

**FIG. 1.**
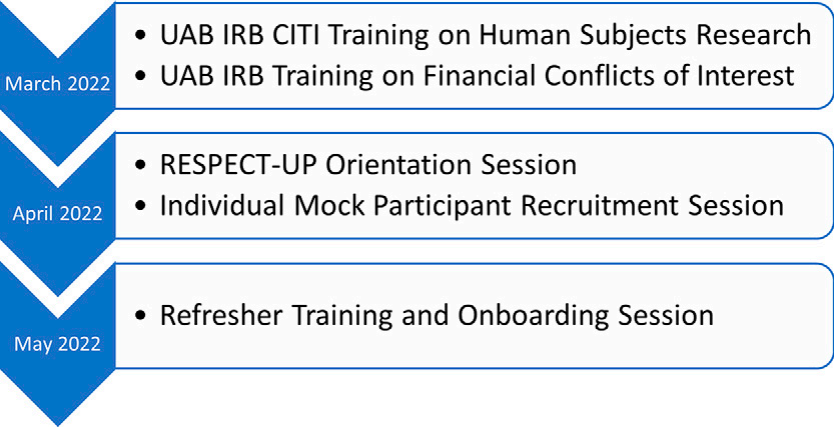
Training of Community Engagement Coordinators: Timeline and Milestones.

The CECs underwent structured training as research coordinators, which was delivered in 3 group sessions and 1 individual session conducted March–May 2022 (Fig. 1). The didactic training included topics on human subjects research, informed consent, financial conflicts of interest, and conflict resolution. To mitigate the risk of coercion, CECs were extensively trained on the importance of voluntary participation and participants’ rights to withdraw from a research study. CECs then completed the formal training courses required by the UAB Institutional Review Board—Collaborative Institutional Training Initiative (CITI) training on research with human subjects and UAB training on financial conflicts of interest—and received certificates of completion. Next, we organized two orientation sessions about the RESPECT-UP study, focusing specifically on study eligibility, survey administration, data integrity and quality, and the use of the study-specific recruitment and data collection tools described below. Following the orientation sessions, each CEC was required to complete a mock participant recruitment session with members of the community engagement team to ensure that all aspects of recruitment, consent, and survey administration were fully mastered. A refresher training and onboarding session was conducted immediately prior to the beginning of study recruitment.

### Materials Provided to CECs

To facilitate participant recruitment and data collection, we provided each CEC with recruitment materials and a tablet (Apple iPad) equipped with the tools described below.

#### Recruitment materials

**FIG. 2.**
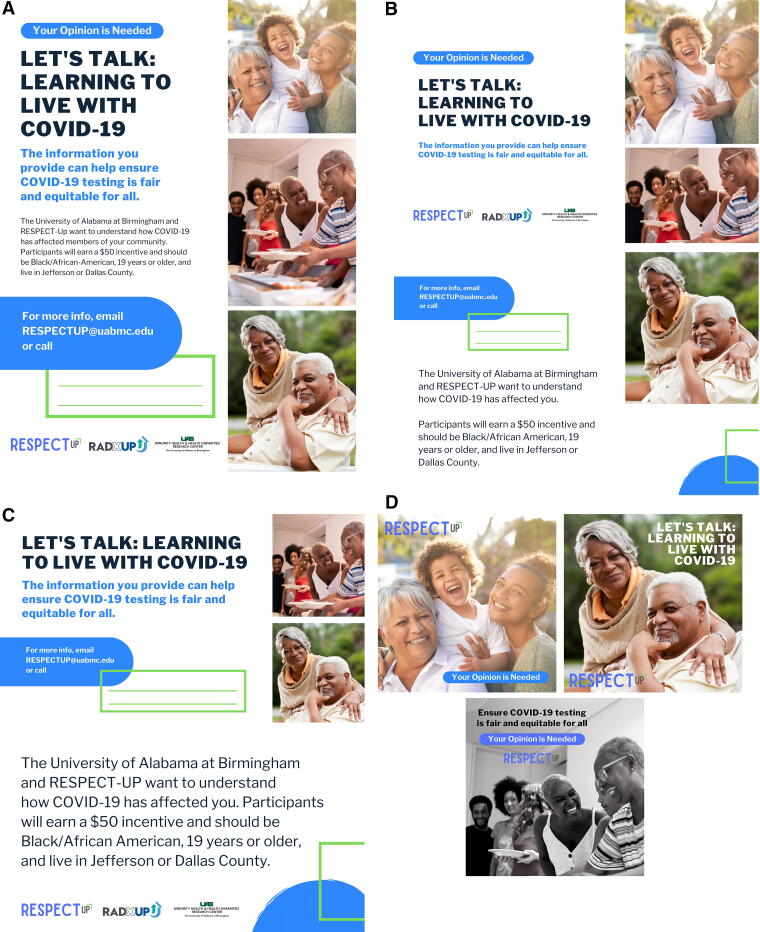
Recruitment materials used by Community Engagement Coordinators (CEC).

We developed a variety of printed and online RESPECT-UP recruitment materials (Fig. 2): printed 8.5 × 11 flyers (Fig. 2A), printed 4 × 6 postcards, (Fig. 2B), printed 2 × 3 business cards (Fig. 2C), and online social media assets (Fig. 2D). All recruitment materials were customizable, allowing CECs to insert their own name and contact information. Recruitment materials were developed with input from the CECs and were approved by the CAB. The CAB also facilitated recruitment by disseminating recruitment materials to their members or constituents.

#### Interactive mapping tool

Tablets were equipped with an interactive mapping tool developed by our study team. The tool allowed CECs to easily determine whether a potential participant was eligible for recruitment (resident of a Census tract in the top 10% of the Social Vulnerability Index) based on their street address.

#### Survey form

Each tablet included a consent form and a survey form linked to the study REDCap database. These tools allowed CECs to document consent and enter survey data directly into the REDCap database.

### CEC Study Activities

**FIG. 3.**
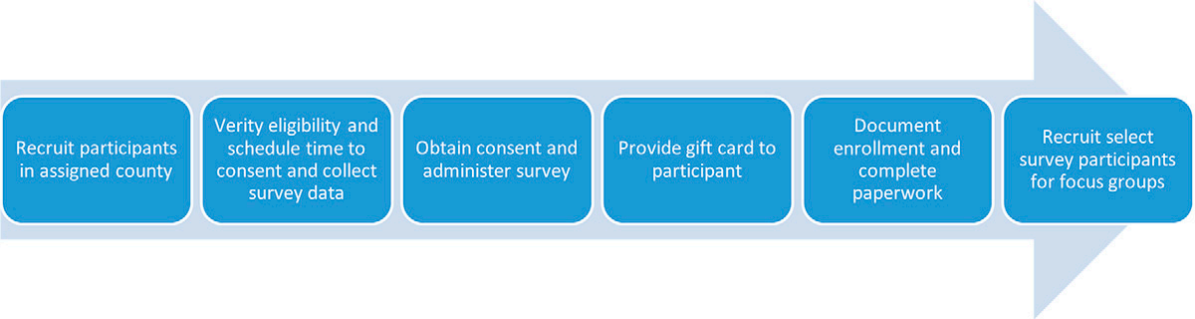
Workflow of Community Engagement Coordinator (CEC) study activities.

CECs were tasked with recruitment, consenting, and data collection in Jefferson (urban) and Dallas (rural) Counties, with a planned recruitment of 300 participants (240 urban and 60 rural) from eligible Census tracts in each county. Figure 3 illustrates the workflow of CEC study activities: (a) recruitment of potential participants from assigned county; (b) verification of eligibility and scheduling a telephone or in-person meeting (in a public place); (c) obtaining consent and administering the survey; (d) providing a $50 gift card to the participant; and (e) documenting enrollment and completing paperwork. CECs were trained to keep communication logs with all recruitment attempts and outcomes. Two partnering community-based organizations (CBOs)—Tiara Straightened in Jefferson County and the Black Belt Community Foundation in Dallas County—served as study fiscal agents for each respective county and were responsible for issuing payments to the CECs. CECs were compensated based on the number of completed surveys ($40 per survey).

CECs were supervised by assigned study personnel. CECs provided information about study progress, successes, and challenges in weekly individual sessions with their study supervisor and bi-weekly group sessions with the entire study team and all CECs. The individual sessions were used to provide support specific to each CEC, while the group sessions allowed CECs to share with each other experiences, troubleshoot challenges, establish camaraderie, and create a sense of belonging to the study research team.

After survey data collection was completed, CECs recruited and scheduled participants in focus groups from a list of survey respondents stratified by select study criteria. Focus group participants were compensated $50; CECs were compensated a one-time payment of $500 each for contacting, scheduling, and making reminder calls for focus group participants.

### CEC Experiences

After data collection was completed, we conducted individual semi-structured qualitative interviews with CECs to gain a deeper insight about their experience with the study. Interviews were conducted virtually via the meeting platform Zoom. An IRB-approved discussion guide included topics related to the training process, the study materials and resources, the recruitment of participants, the overall experience with the study, and areas for improvement in future community-engaged research. For data analysis, we used a framework-guided rapid analysis (RA) approach to identify high-level themes.^27,28^ A structured templated summary table was developed before the interviews. The table was categorized by high-level domains outlined in the interview guide and subcategorized by specific questions within each domain from the interview guide. Data, including illustrative quotes, were extracted from verbatim transcripts and entered in the templated summary table by researchers for review.

## Results

### CEC Recruitment and Training

A total of 10 lay individuals from the targeted communities were recruited and trained as CECs: 8 from Jefferson County (urban) and 2 from Dallas County (rural), 8 female and 2 male. CEC recruitment was completed in 2 rounds, with 7 individuals recruited in the first and 3 in the second round. Of 10 trained CECs, 6 remained active until the end of the study (4 in Jefferson, 2 in Dallas) and 4 stopped study activities before completion (4 in Jefferson, 0 in Dallas). All who withdrew early requested to be considered for future studies. Reasons for early withdrawal included sickness, loss of employment, and other personal or family circumstances.

All 10 CECs completed 5 h of instructor-led training, CITI Human Subjects training, UAB Financial Conflict of Interest training, an individual mock participant recruitment session, and a group refresher and onboarding session. CECs received an estimated average of 11 h of training, with some CECs undergoing additional refresher training as requested.

### Survey Recruitment and Data Collection

CECs used 1,200 supplied copies of customizable recruitment materials and the study interactive mapping tool to reach a planned recruitment of 300 participants (240 urban and 60 rural). In total, CECs attempted 313 survey administrations and completed 303 of them. Surveys were administered either in-person and via telephone, and each session lasted approximately 1 h.

CECs used a snowball recruitment method by receiving referrals from trusted contacts in the target communities. The mean number of participants recruited by a CEC was 60 (range 43–91, SD 1.3) in Jefferson County and 30 (range 30–31, SD 0) in Dallas County. CECs were given a recruitment goal of six surveys per week. To meet the study timeline, recruitment goals were increased to 12 surveys per week, with a $100 bonus for each survey above the weekly goal. Planned recruitment and data collection were completed in 11 weeks. When accounting for survey skip patterns and branching logic, the rate of missing data was below 3%.

### Focus Group Recruitment

We provided CECs with a list of 169 potential participants in focus groups from survey respondents stratified by daily discrimination scores and COVID-19 testing attitude scores. The number of potential participants was divided evenly between the remaining CECs (approximately 60 per CEC in Jefferson County and 40 per CEC in Dallas County). The planned enrollment was 50 participants in 8 focus groups. CECs completed recruitment in 2 weeks and recruited a total of 53 focus group participants.

### CEC Qualitative Interviews

Four of the six CECs who remained active in the study participated in interviews. The rapid analysis identified three common themes: (1) Gaining trust, (2) leveraging of community relationships, and (3) establishing personal connection with participants. CECs reported that trust with potential participants was established quickly when CECs identified as a member of the community as well as a representative of the research team. One CEC shared, “You know, when people know you, they trust you. They know when I tell them about (research) and what it means, that I’m not lying. I’m telling them the truth and telling them what it’s really about.” CECs reported that to reach their target enrollment goals, they leveraged longstanding community relationships through faith-based organizations, family relationships, and previous work or educational connections. CECs also stated that the use of in-person recruitment and survey administration helped them establish a personal connection with responders, which allowed for flexibility in participant engagement and contributed to high data quality. Some CECs shared that their work on the project gave them a better understanding of the research process and allowed them to advocate for the importance of research and reassure community members of their safety when engaging in biomedical research. When discussing the personal lessons learned working in research, one CEC stated, “I know more about it now and I can help other people know more too.”

## Discussion

This RESPECT-UP project aimed to understand social, ethical, and behavioral factors surrounding COVID-19 testing in vulnerable urban and rural African American communities in Alabama. To overcome widespread research mistrust in this population, we trained lay community members and used them as research personnel tasked with participant recruitment and data collection. Beyond trust, this approach helped address unique challenges posed by recruitment of participants from geographically dispersed areas and distinct community characteristics (rural and urban), where a “one-size-fits-all” recruitment may have underestimated the heterogeneity of African American communities in the state. Our strategy proved successful as evidenced by efficient enrollment and high-quality data collection. In addition, the engagement of CECs expanded the community infrastructure available for future studies and, based on CEC feedback, may have promoted community support for future research and public health interventions. Our experience also revealed challenges that need to be addressed to maximize mutually beneficial academic-community collaborations.

### Successes

CECs were extremely effective as research personnel. They were able to meet and surpass planned enrollment goals in less time than anticipated and collect data with superior quality.^29–31^ Several factors contributed to this success. CECs shared that it was easier for them to approach and gain the trust of fellow community residents. They also reported satisfaction from interacting with study participants and described building deeper connections in their own community. These findings corroborate previous research, which has found that African American study participants prefer more personal recruitment process that is based in a community setting and carried out by culturally matched research personnel.^32,33^ For example, some CECs organized or leveraged community events, such as sporting events and faith-based outreach, to optimize resources and maximize recruitment. CECs approached community members in a culturally appropriate and respectful manner, which fostered trust and understanding. Other factors that facilitated success, according to CECs, were the snowball recruitment method, appropriate incentives for study participation, and bonuses to CECs for exceeding weekly recruitment goals. It is important to note that project goals were shared openly with CECs to facilitate transparency and accountability. CECs were given a clear job description, project outcomes, and benchmarks. For task performance, CECs received prompt, accurate, and easily understood information.

Positive experiences of engaging community members as data collectors has been noted in other populations during the COVID-19 pandemic, including refugee populations.^34^

### Challenges

Our experience showed that work or family responsibilities often limit the ability of CECs to meet study responsibilities in a timely manner. A paid model of community engagement has been found to increase the amount of work seen as reasonable by community health workers.^18^ Applying this model, we introduced a weekly incentive for exceeding recruitment goals and observed a rapid increase in participant enrollment.

A particular challenge reported by CECs was the collection of Tier 1 common data elements (CDEs) required by the funding organization. During weekly meetings, CECs reported that survey participants expressed concern when asked to share personal information related to height, weight, pregnancy status, or sexual orientation. While hesitation to share such sensitive information can be expected among most research participants, historical mistrust of research in the African American community further compounds the problem.^6,14–16^ In-person survey administration by trained staff can reduce the rate of low-quality responses.^19,20^ Therefore, CECs were trained to explain why these questions are important and reassure participants of the confidentiality of their answers while emphasizing that participation is voluntary. CECs received response scripts and practiced quick and effective explanations for the collection of sensitive data. Our experience shows that training with role-playing elements, scripts that clarify potentially confusing terms, and careful explanation of the research goals is beneficial for mitigating the risk of incomplete or inaccurate responses. Overall, CECs were able to leverage their community status and personal experiences and identity as African Americans to gain the trust of respondents, which resulted in high data quality.

### Opportunities and Future Directions

Training and engaging lay community members as paid research staff is a powerful tool for building a community research capacity. Investing in vulnerable African American communities by training and employing their members as research personnel creates a viable research infrastructure for future studies. Expanding the knowledge of community leaders of the research process and facilitating positive research experiences by community members can help rebuild the African American community’s trust in biomedical science and promote support for future public health interventions. The RESPECT-UP experience showed that trained community members can be invaluable members of the research team. The impact of this approach for community awareness and acceptance of research as well as for long-term community health outcomes needs to be assessed.

## Declaration of Interest

The authors declare no potential conflicts of interest with respect to the research, authorship, and/or publication of this article.

## Human Participation Protection

This study was approved by the Institutional Review Board of the University of Alabama at Birmingham (UAB), protocol # IRB-300008595.

## Abbreviations Used

CAB: Community Advisory Board
CBO: Community-Based Organization
CDC: Centers for Disease Control and Prevention
CDEs: Common Data Elements
CEC: Community Engagement Coordinators
CITI: Collaborative Institutional Training Initiative
IRB: Institutional Review Board
RADx-UP: Rapid Acceleration of Diagnostics (RADx^®^) Underserved Populations
REDCap: Research Electronic Data Capture
RESPECT-UP: Reducing Ethical and Social Prejudicial Effects of COVID-19 Testing in Underserved Populations
SD: Standard Deviation
UAB: University of Alabama at Birmingham

## References

[1] Centers for Disease Control and Prevention. COVID Data Tracker. CDC. Updated July 13, 2023. [Last accessed: July 23, 2023]. Available from: https://covid.cdc.gov/covid-data-tracker/#vaccination-demographics-trends

[2] Williams DR, Rucker TD. Understanding and addressing racial disparities in health care. Health Care Financ Rev 2000;21(4):75–90. 11481746 PMC4194634

[3] Statistics NCfH. Health, United States 2018. 2018. Available from: https://www.cdc.gov/nchs/data/hus/hus18.pdf 31944640

[4] Wright S. Trust and trustworthiness. Philosophia 2010;38(3):615–627.

[5] Nydegger LA, Hill MJ. Examining COVID-19 and HIV: The impact of intersectional stigma on short- and long-term health outcomes among African Americans. Int Soc Work 2020/09/01 2020;63(5):655–659; doi: 10.1177/0020872820940017 38323072 PMC10846888

[6] Moore AD, Hamilton JB, Knafl GJ, et al. The influence of mistrust, racism, religious participation, and access to care on patient satisfaction for African American men: The North Carolina-Louisiana Prostate Cancer Project. J Natl Med Assoc 2013;105(1):59–68; doi: 10.1016/s0027-9684(15)30086-9 23862297

[7] Mayberry RM, Mili F, Ofili E. Racial and ethnic differences in access to medical care. Med Care Res Rev 2000;57(Suppl 1):108–145; doi: 10.1177/1077558700057001s06 11092160

[8] Institute of Medicine. Unequal Treatment: confronting racial and ethnic disparities in health care. The National Academies Press; 2003. 25032386

[9] House J. Understanding and Reducing Socioeconomic and Racial/Ethnic Disparities in Health. In: Smedley B., ed. Promoting Health: Intervention Strategies from Social and Behavioral Research. National Academies Press; 2000. Institute of Medicine (US) Committee on Capitalizing on Social Science and Behavioral Research to Improve the Public’s Health. 25057721

[10] Colen C, Ramey D, Cooksey E, et al. Racial disparities in health among nonpoor African Americans and Latinos: The role of acute and chronic discrimination. Soc Sci Med 2018;199:167–180; doi: 10.1016/j.socscimed.2017.04.051 28571900 PMC5673593

[11] Washington H. Medical Apartheid: The Dark History of Medical Experimentation on Black Americans from Colonial Times to the Present. DoubleDay Books; 2006.

[12] Williams DR, Cooper LA. COVID-19 and health equity—a new kind of “herd immunity. JAMA 2020;323(24):2478–2480; doi: 10.1001/jama.2020.8051 32391852

[13] Bateman LB, Schoenberger Y-MM, Hansen B, et al. Confronting COVID-19 in under-resourced, African American neighborhoods: A qualitative study examining community member and stakeholders’ perceptions. Ethnicity &Amp; Health 2021;26(1):49–67; doi: 10.1080/13557858.2021.1873250 PMC7875151 33472411

[14] Cyril S, Smith BJ, Possamai-Inesedy A, et al. Exploring the role of community engagement in improving the health of disadvantaged populations: A systematic review. Glob Health Action 2015;8(1):29842; doi: 10.3402/gha.v8.29842 26689460 PMC4685976

[15] Wilson D, Neville S. Culturally safe research with vulnerable populations. Contemp Nurse 2009;33(1):69–79; doi: 10.5172/conu.33.1.69 19715497

[16] Racial/Ethnic Disparities in pregnancy-related deaths - United States, 2007–2016. Accessed [Last accessed: July 14, 2023]. Available from: https://www.cdc.gov/reproductivehealth/maternal-mortality/disparities-pregnancy-related-deaths/infographic.html 10.15585/mmwr.mm6835a3 PMC6730892 31487273

[17] Farmer N, Osei Baah F, Williams F, et al. Use of a community advisory board to build equitable algorithms for participation in clinical trials: A protocol paper for HoPeNET. BMJ Health Care Inform 2022;29(1):e100453; doi: 10.1136/bmjhci-2021-100453 PMC8860013 35185011

[18] Schulz AJ, Parker EA, Israel BA, et al. Addressing social determinants of health through community-based participatory research: The East Side Village Health Worker Partnership. Health Educ Behav 2002;29(3):326–341; doi: 10.1177/109019810202900305 12038742

[19] Halladay JR, Donahue KE, Sleath B, et al. Community Advisory Boards Guiding Engaged Research Efforts within a Clinical Translational Sciences Award: Key Contextual Factors Explored. Prog Community Health Partnersh 2017;11(4):367–377; doi: 10.1353/cpr.2017.0044 29332850 PMC8585511

[20] Robinson RG. Community Development Model for Public Health Applications: Overview of a Model to Eliminate Population Disparities. Health Promot Pract 2005;6(3):338–346; doi: 10.1177/1524839905276036 16020628

[21] Hatch J, Moss N, Saran A, et al. Community Research: Partnership in Black Communities. Am J of Prev Med 1993;9(6 Supplement):27–31; doi: 10.1016/S0749-3797(18)30662-7 8123284

[22] Greiner K, Friedman D, Adams S, et al. Effective recruitment strategies and community-based participatory research: Community networks program centers’ recruitment in cancer prevention studies. Cancer Epidemiol Biomarkers Prev 2014;23(3):416–423; doi: 10.1158/1055-9965.EPI-13-0760 24609851 PMC3971731

[23] Fouad MN, Partridge E, Dignan M, et al. A community-driven action plan to eliminate breast and cervical cancer disparity: Successes and limitations. J Cancer Educ 2006;21(1):s91–100; doi: 10.1207/s15430154jce2101s_16 17020510

[24] Israel BA. Social networks and social support: Implications for natural helper and community level interventions. Health Educ Q 1985;12(1):65–80. 3980242 10.1177/109019818501200106

[25] Rosenthal EL, Wiggins N, Ingram M, et al. Community health workers then and now: An overview of national studies aimed at defining the field. J Ambul Care Manage 2011;34(3):247–259; doi: 10.1097/JAC.0b013e31821c64d7 21673523

[26] Centers for Disease Control and Prevention. CDC/ATSDR Social Vulnerability Index. 2023. Available from: https://www.atsdr.cdc.gov/placeandhealth/svi/index.html

[27] Nevedal AL, Reardon CM, Opra Widerquist MA, et al. Rapid versus traditional qualitative analysis using the Consolidated Framework for Implementation Research (CFIR). *Implementation*. Implement Sci 2021;16(1):67; doi: 10.1186/s13012-021-01111-5 34215286 PMC8252308

[28] Gale RC, Wu J, Erhardt T, et al. Comparison of rapid vs in-depth qualitative analytic methods from a process evaluation of academic detailing in the Veterans Health Administration. Implement Sci 2019;14(1):11; doi: 10.1186/s13012-019-0853-y 30709368 PMC6359833

[29] Dong Y, Peng C-YJ. Principled missing data methods for researchers. Springerplus 2013;2(1):222; doi: 10.1186/2193-1801-2-222 23853744 PMC3701793

[30] Kang H. The prevention and handling of the missing data. Korean J Anesthesiol 2013;64(5):402–406; doi: 10.4097/kjae.2013.64.5.402 23741561 PMC3668100

[31] Schafer JL. Multiple imputation: A primer. Stat Methods Med Res 1999;8(1):3–15; doi: 10.1177/096228029900800102 10347857

[32] Breland-Noble AM, Bell CC, Burriss A, et al. The AAKOMA Project Adult Advisory Board. The significance of strategic community engagement in recruiting African American Youth &amp; Families for clinical research. J Child Fam Stud 2012;21(2):273–280; doi: 10.1007/s10826-011-9472-1 22984337 PMC3439824

[33] George S, Duran N, Norris K. A systematic review of barriers and facilitators to minority research participation among African Americans, Latinos, Asian Americans, and Pacific Islanders. Am J Public Health 2014;104(2):e16–e31; doi: 10.2105/ajph.2013.301706 PMC3935672 24328648

[34] Disney L, Ahmed R, Carnes S. advancing community-based participatory research during the COVID-19 Pandemic: A methods commentary on the lessons learned from working with community data collectors on a Refugee Health Disparities Study. J Health Commun 2023;28(sup1):2–6; doi: 10.1080/10810730.2023.2187102 37390015

